# Patient experience in the treatment of metastatic castration-resistant prostate cancer: state of the science

**DOI:** 10.1038/pcan.2015.42

**Published:** 2016-02-02

**Authors:** N Nussbaum, D J George, A P Abernethy, C M Dolan, N Oestreicher, S Flanders, T B Dorff

**Affiliations:** 1 Department of Genitourinary Cancers, Duke Cancer Institute, Durham, NC, USA; 2 Flatiron Health, Inc., New York, NY, USA; 3 CMD Consulting, Inc., Sandy, UT, USA; 4 Department of Clinical Pharmacy, University of California San Francisco, San Francisco, CA, USA; 5 Health Economics and Clinical Outcomes Research, Astellas Pharma Global Development, Inc., Northbrook, IL, USA; 6 USC Norris Cancer Hospital, Keck School of Medicine, University of Southern California, Los Angeles, CA, USA

## Abstract

**Background:**

Contemporary therapies for metastatic castration-resistant prostate cancer (mCRPC) have shown survival improvements, which do not account for patient experience and health-related quality of life (HRQoL).

**Methods:**

This literature review included a search of MEDLINE for randomized clinical trials enrolling ⩾50 patients with mCRPC and reporting on patient-reported outcomes (PROs) since 2010.

**Results:**

Nineteen of 25 publications describing seven treatment regimens (10 clinical trials and nine associated secondary analyses) met the inclusion criteria and were critically appraised. The most commonly used measures were the Functional Assessment of Cancer Therapy-Prostate (*n*=5 trials) and Brief Pain Inventory Short Form (*n*=4 trials) questionnaires. The published data indicated that HRQoL and pain status augmented the clinical efficacy data by providing a better understanding of treatment impact in mCRPC. Abiraterone acetate and prednisone, enzalutamide, radium-223 dichloride and sipuleucel-T offered varying levels of HRQoL benefit and/or pain mitigation versus their respective comparators, whereas three treatments (mitoxantrone, estramustine phosphate and docetaxel, and cabazitaxel) had no meaningful impact on HRQoL or pain. The main limitation of the data were that the PROs utilized were not developed for use in mCRPC patients and hence may not have comprehensively captured symptoms important to this population.

**Conclusions:**

Recently published randomized clinical trials of new agents for mCRPC have captured elements of the patient experience while on treatment. Further research is required to standardize methods for measuring, quantifying and reporting on HRQoL and pain in patients with mCRPC in the clinical practice setting.

## Introduction

Prostate cancer is the third most commonly diagnosed malignancy in the United States (after breast and lung), with an estimated 220 800 new cases and 27 540 deaths in 2015.^1^Most patients present with localized disease and undergo initial surgical and/or radiological therapy, with concomitant or subsequent use of androgen deprivation therapy (ADT). Generally, PSA level should be <0.5 ng ml^−1^ after radiation therapy and <0.2 ng ml^−1^ after a radical prostatectomy,^2^ and occurrence of two consecutive PSA level elevations is often considered biochemical recurrence or progression to stage D1.5 disease. Biochemical recurrence develops in ≈10% of low-risk and up to 60% of high-risk prostate cancer patients after external beam radiation therapy^3, 4, 5, 6^ and in 20–30% of patients after radical prostatectomy^7, 8, 9^ despite use of ADT.

Once prostate cancer has become metastatic, ADT is deployed and is highly effective, eliciting a response in most cases; however, resistance inevitably develops, resulting in transition to a lethal castration-resistant phenotype, affecting 10–20% of prostate cancer patients within 5 years,^10^ and the death of >50% of patients within 3 years with historical standard therapies.^11, 12, 13, 14, 15^ This end of the disease continuum is termed metastatic castration-resistant prostate cancer (mCRPC), defined by cancer progression despite a testosterone level of <50 ng dl^−1^ (<1.7 nmoll^−1^).^16^

The natural history of mCRPC can involve worsening symptomatology represented by a progressive decline in health-related quality of life (HRQoL) and worsening pain,^10^ where HRQoL is considered a multidomain phenomenon capturing an individual's perceived mental, emotional, physical and social well-being over time.^17, 18^ The first treatments approved by the US Food and Drug Administration for mCRPC management focused on the palliative benefits of pain control achieved by mitoxantrone, strontium and samarium.^19, 20, 21^ In 2004, docetaxel became standard of care after two phase III trials demonstrated a survival benefit over mitoxantrone.^12, 13^ Data from one of these trials^13^ showed that global HRQoL improved from baseline to 6 months in patients receiving docetaxel despite similar rates of pain relief in both groups,^22^ suggesting that pain relief is only a component of HRQoL in mCRPC, as fatigue and physical function (upon which pain can have an impact) are also major contributors. Certainly, asymptomatic patients are more likely to have worsening HRQoL after cytotoxic chemotherapy treatment,^23^ and this risk must be weighed against potential benefits.

Since 2010, a fundamental shift has occurred in the mCRPC treatment landscape with the arrival of immunotherapy (sipuleucel-T (sip-T)), agents targeting androgen signaling (abiraterone acetate and enzalutamide), and a bone-targeting radiopharmaceutical (radium-223 dichloride), which extend survival when utilized before or after docetaxel chemotherapy.^24, 25, 26, 27, 28, 29, 30, 31^ Median overall survival (OS) among patients with nonvisceral mCRPC who received immunotherapy with sip-T was 25.8 versus 21.7 months in the placebo group.^26^ In patients with mCRPC before and after chemotherapy, respectively, targeted therapy with abiraterone acetate plus prednisone (OS, 34.7 vs 30.3 months and 15.8 vs 11.2 months),^25, 31^ enzalutamide (OS, 32.4 vs 30.2 months and 18.4 vs 13.6 months),^24, 30^ and radium-223 dichloride (OS, 16.1 vs 11.5 months and 14.4 vs 11.3 months)^32^ all increased OS relative to control. Additional cytotoxic therapy with cabazitaxel was found to extend OS (15.1 vs 12.7 months) in men whose mCRPC had progressed after docetaxel therapy, when compared with the prior palliative standard of mitoxantrone.^33^ The life-extending noncytotoxic therapies in particular have potential to have a favorable impact on patients' HRQoL and pain and may strike a better balance between cancer control and toxicity.

In response to the Prostate Cancer Clinical Trials Working Group 2 proposed principles of conduct for phase II and III mCRPC trials, the clinical trials of these new therapies evaluated patient-reported outcomes (PROs) to ensure that the overall efficacy and safety profiles of new therapies reflect patient experience and perceptions.^16, 34^ The US Food and Drug Administration defines a PRO as ‘any report of the status of a patient's health condition that comes directly from the patient, without interpretation of the patient's response by a clinician or anyone else.'^17^ PRO instruments typically include information about HRQoL, symptoms, function, satisfaction with care or symptoms, adherence to prescribed medications or other therapy, and perceived value of treatment.^18^ In the prostate cancer setting, multiple instruments have included specific symptoms relevant to the disease (for example, urinary control and hot flashes), the most widely used being the multidimensional Functional Assessment of Cancer Therapy-Prostate (FACT-P) questionnaire, which indicates a worsening or improving HRQoL when the total score changes by at least 6–10 points on a 0–156 scale.^35^

Here we review PRO data from clinical trials of patients with mCRPC reported since 2010 in order to contextualize the overall impact of new treatment modalities from the patient's perspective, and in so doing, guide patient-centered care, clinical decision-making, and health policy decisions.

## Patients and Methods

To review clinical trials reporting PROs in patients with mCRPC, we conducted two separate searches of the US National Library of Medicine National Institutes of Health Medline database for full articles published between January 2010 and April 2015. We first created a database of all randomized, controlled trial articles in mCRPC using the following Boolean search term strategy: (prostate cancer) AND (castration-resistant OR hormone-refractory OR androgen independence) AND (randomized OR randomised). A second search was conducted as follows: (prostate cancer) AND (health-related quality of life OR HRQoL OR QoL) OR pain OR fatigue OR weight loss. Both searches were limited to articles published in English and combined to create the full data set of potentially eligible articles. Additional references were identified from bibliographies of published articles.

We screened the title and abstract of each retrieved article for relevance against predefined inclusion criteria: clinical studies in mCRPC with a sample size ⩾50. We qualitatively reviewed full text articles for final inclusion and assessment based on the following endpoints of interest: change from baseline in PRO scores; time to improvement or deterioration in a PRO measure; and proportion of patients with improvement or deterioration in a PRO measure.

## Results

We identified 26 publications meeting our predefined inclusion criteria. Six were excluded because the therapies described are not used in standard clinical practice.^36, 37, 38, 39, 40, 41^ Nineteen publications describing seven treatment regimens (ten clinical trials and ten associated secondary analyses) were reviewed (Table 1).

### PRO Instruments

Across the 10 clinical trials, 7 different patient-completed questionnaires measuring HRQoL and/or pain were used. All PROs had demonstrated reliability, had been subject to validation processes, were responsive to change in health state, and had well-established psychometric characteristics although the Pain Index has not been subject to same validation processes as far as we are aware (Table 2). In the identified trials, HRQoL instruments were most often used along with a separate pain instrument. The two most commonly used PROs were the FACT-P questionnaire (used in five of the trials),^42, 43, 44^ and the Brief Pain Inventory (BPI) Short Form (SF; used in four of the trials).^45^ Three of the trials did not use an HRQoL instrument and only used pain instruments.^33, 46, 47^ These trials and one other collected data regarding use of analgesics, specifically opiate medications.^46, 47, 48, 49^ Conversely, two trials did not use a dedicated pain instrument and used an HRQoL instrument only.^50, 51^ The PREVAIL study (Table 1) used two complementary tools to evaluate HRQoL: the prostate-cancer-specific FACT-P questionnaire and the generic EQ-5D questionnaire.^52^ Both the PREVAIL and AFFIRM studies reported on pain using the FACT-P prostate cancer subscale (PCS) pain-related items, which complemented utilization of the BPI-SF.^52^ Patient-reported fatigue was reported in three studies: one utilized the Brief Fatigue Inventory (COU-AA-301)^53^ and the others utilized the fatigue symptom scale questionnaire of the European Organization for Research and Treatment of Cancer Quality of Life Questionnaire (EORTC QLQ)-C30.^49, 51^ Studies reporting FACT-P total and PCS scores included items addressing fatigue but, to date, no results have been published on fatigue domains of FACT-P scores specifically.

### Treatment-related changes in PROs

Most studies reported time-to-event analyses (for example, time to improvement or deterioration in FACT-P total score) by use of Kaplan–Meier survival analysis and/or the proportion of patients with clinically meaningful improvement in a PRO score. Changes from baseline in mean scores for a particular PRO measure were not reported routinely.

#### Abiraterone acetate

Two registrational, placebo controlled, phase III studies of abiraterone acetate in mCRPC examined HRQoL and pain in patients progressing after docetaxel chemotherapy (COU-AA-301) and before docetaxel chemotherapy (COU-AA-302) despite ongoing ADT.^29, 54^ Patient compliance rates with PRO questionnaires were high during both studies.^28, 53, 55, 56, 57^

**Figure 1:**
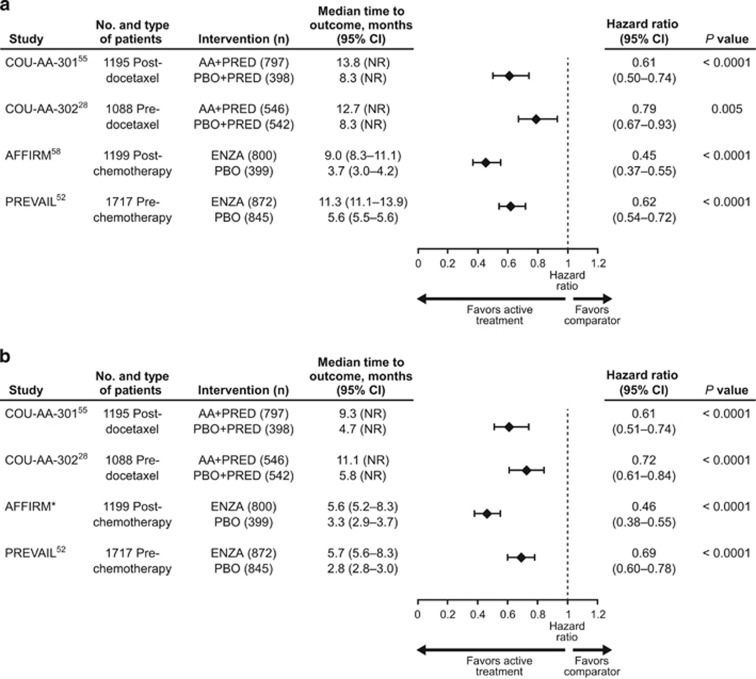
Risk for a clinically meaningful deterioration in (**a**) FACT-P total score and (**b**) FACT-P PCS score. AA, abiraterone acetate; CI, confidence interval; ENZA, enzalutamide; FACT-P, Functional Assessment of Cancer Therapy-Prostate total score; NR, not reported; PBO, placebo; PCS, prostate cancer subscale; PRED, prednisone. *AFFIRM FACT-P PCS score data were taken from Cella D *et al.*^63^ and used with permission.

**Figure 2:**
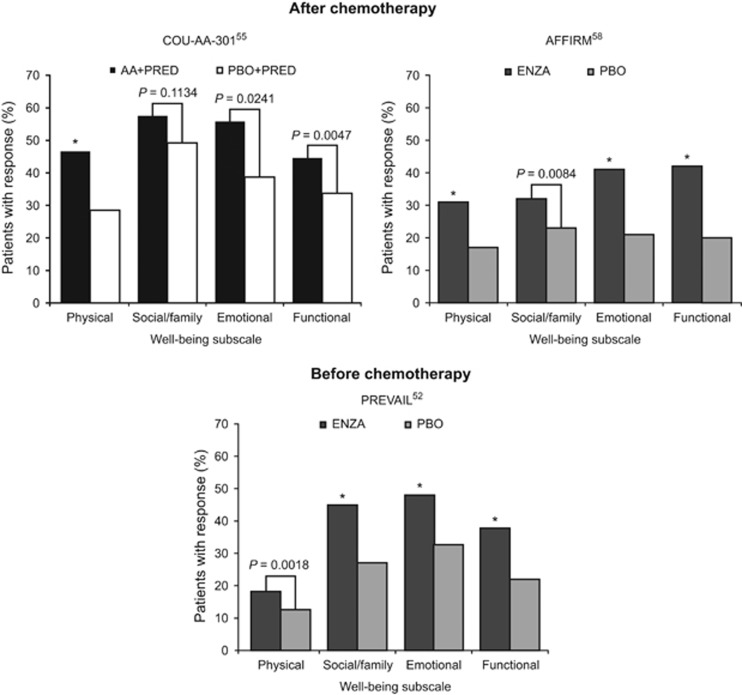
Proportion of patients with metastatic castration-resistant prostate cancer reporting a clinically meaningful improvement on the FACT-P subscale well-being scores after receipt of noncytotoxic therapies. AA, abiraterone acetate; ENZA, enzalutamide; FACT-P, Functional Assessment of Cancer Therapy-Prostate; PBO, placebo; PRED, prednisone. *P<0.0001 vs comparator.

In COU-AA-301, patients had mean baseline FACT-P total scores approximating 108 of the maximum possible score of 156, indicating that these patients had a moderate level of HRQoL impairment.^55^ Changes in estimated FACT-P total score from baseline to week 112 favored the abiraterone acetate plus prednisone arm over the placebo plus prednisone arm throughout the study (104 to 50 points vs 104 to 30 points).^55^ Median times to deterioration in FACT-P total score and PCS, as defined in Table 2, were delayed in the abiraterone acetate plus prednisone arm relative to the placebo plus prednisone arm (Figure 1), as were times to deterioration on all other FACT-P subscales with the exception of social/family well-being.^55^ Additionally, median time to improvement in fatigue intensity (59 days vs 194 days; *P*=0.0155) was shortened in the abiraterone acetate plus prednisone arm.^53^ Greater proportions of patients in the abiraterone acetate plus prednisone arm than placebo plus prednisone arm reported improvements in FACT-P total and PCS scores (Table 3), as well as improvement on all FACT-P subscales (with the exception of social/family well-being; Figure 2). Similarly, a Brief Fatigue Inventory responder analysis of patients with clinically meaningful fatigue at baseline favored the abiraterone acetate plus prednisone arm (fatigue intensity, 58% vs 40% *P*=0.0001; fatigue interference, 55% vs 38% *P*=0.0075).^53^

Patients enrolled in COU-AA-301 were considered mildly symptomatic based on a median BPI-SF question 3 score of 3 (range 0–10, with higher scores indicating a greater severity of worst pain in the last 24 h).^56^ The BPI-SF pain data from COU-AA-301 showed a clear benefit in the abiraterone acetate plus prednisone arm, as evidenced by longer median times to first deterioration in worst pain and pain interference, where progression on both pain outcomes was confirmed at two consecutive follow-up visits (Table 4). This study also measured palliation of worst pain, but only among those with clinically significant worst pain at baseline defined as a score of ⩾4 on BPI-SF question 3. Worst pain intensity palliation was defined as two consecutive follow-up visits (⩾4 weeks apart) at which the worst pain intensity score was ⩾30% lower than that at baseline without an increase in analgesic use, whereas pain interference palliation was defined as a decrease in mean pain interference score of ⩾1.25 points compared with baseline at two consecutive follow-up visits. Median time to palliation in worst pain intensity (5.6 vs 13.7 months; hazard ratio (HR), 1.68; 95% confidence interval (CI), 1.20–2.34; *P*=0.0018) and interference (1.06 vs 3.7 months; HR, 1.89; 95% CI, 1.31–2.74; *P*=0.0004) was shorter in the abiraterone acetate plus prednisone arm than placebo plus prednisone arm. A greater proportion of abiraterone acetate plus prednisone than placebo plus prednisone recipients reported clinically meaningful palliation of worst pain intensity (45% vs 29% *P*=0.0005) and pain interference (60% vs 38% *P*=0.0002). Median duration of worst pain intensity palliation was also longer in the abiraterone acetate plus prednisone arm than placebo plus prednisone arm (4.2 vs 2.1 months; *P*=0.0056).^56^

In COU-AA-302, baseline FACT-P total scores averaged ≈122, indicating a patient population with a better HRQoL than the COU-AA-301 population.^57^ Patients who received abiraterone acetate plus prednisone had lower risks for and longer median times to first deterioration in FACT-P total score and PCS scores than patients who received placebo plus prednisone (Figure 1). A significant difference was also seen in favor of abiraterone acetate plus prednisone regarding time to deterioration on each FACT-P subscale except social/family well-being.^28^ Median time to progression in pain interference with daily activities on the BPI-SF was longer in the abiraterone acetate plus prednisone arm than in the placebo plus prednisone arm, but no statistically significant between-group differences were observed regarding both mean and worst pain intensity (Table 4).^28^ Median time to opiate use for prostate cancer-related pain was delayed with abiraterone acetate plus prednisone relative to placebo plus prednisone (33.4 vs 23.4 months; HR, 0.72; 95% CI, 0.61–0.85).^31^

#### Enzalutamide

The FACT-P and BPI-SF completion rates were high throughout both registrational phase III studies of enzalutamide versus placebo, AFFIRM (following chemotherapy) and PREVAIL (chemotherapy-naive).^24, 30, 52, 58^

In AFFIRM, mean FACT-P total score decreased by 1.5 points with enzalutamide compared with 13.7 points with placebo after 25 weeks (*P*<0.001).^35^ In addition, significant treatment differences at week 25 favoring enzalutamide over placebo were evident for mean changes from baseline across all FACT-P subscale and index scores.^35^ Median times to deterioration in FACT-P total and PCS scores were longer in the enzalutamide arm than placebo arm (Figure 1).^58^ A greater proportion of patients in the enzalutamide arm than placebo arm experienced an improvement in FACT-P total and PCS scores (Table 3) and all FACT-P subscale scores (Figure 2).

In AFFIRM, enzalutamide was associated with change from baseline to week 13 improvements in mean scores of the FACT-P item 4 (that is, ‘I have pain'), BPI-SF pain severity and BPI-SF pain interference (all *P*<0.0001).^58^ Enzalutamide was associated with a 44% reduction in risk for pain progression relative to placebo on FACT-P item 4 in AFFIRM (Table 4).^58^ Of 64 patients (5%) who were evaluable for pain palliation assessments, 22 (45%) of 49 patients receiving enzalutamide reported pain palliation at week 13 versus one (7%) of 15 receiving placebo (difference 38% *P*=0.0079). A smaller proportion of patients had BPI-SF pain progression in the enzalutamide arm than in the placebo arm (28% vs 39% *P*=0.0018).^58^

The PREVAIL patient population had not yet been burdened by significant disease-related symptoms, but nevertheless had mild HRQoL impairment at baseline as evidenced by median baseline FACT-P total scores of 121 (range, 63–156) in the enzalutamide arm and 122 (range, 60–155) in the placebo arm.^52^ Multiple measures of HRQoL and health status favored enzalutamide over placebo, including changes from baseline in FACT-P total (−5.08 vs −10.87; *P*<0.0001), FACT-P PCS (−1.99 vs −3.18; *P*=0.0197) and EQ-5D visual analog scale (VAS; −5.18 vs −9.76; *P*<0.0010) scores measured at week 61.^52^ Median times to deterioration in FACT-P total and PCS scores were longer in the enzalutamide arm than placebo arm (Figure 1), as were median times to deterioration in all other FACT-P subscale scores.^52^ Similar findings in favor of enzalutamide over placebo were detected on the EQ-5D utility index (19.2 months vs 11.1 months; HR, 0.62; 95% CI, 0.52–0.73; *P*<0.0001) and EQ-5D VAS (22.1 months vs 13.8 months; HR, 0.67; 95% CI, 0.56–0.80; *P*<0.0001).^52^

The proportion of patients reporting improvements at any time during the study in FACT-P total (40% vs 23%) and PCS (55% vs 34%) scores (Table 3), as well as EQ-5D utility index (28% vs 16%) and VAS (27% vs 18%) scores, were higher in the enzalutamide than placebo arm (all *P*<0.0001).^52^ Significantly more enzalutamide patients than placebo patients had an improvement at any time during the study in all FACT-P subscale scores (Figure 2).

Mean change-from-baseline scores for BPI-SF severity (0.52 vs 0.79; *P*=0.0025) and interference (0.58 vs 0.99; *P*<0.0001) measured at week 25 favored enzalutamide over placebo.^52^ A lower proportion of enzalutamide patients than placebo patients reported progression of worst pain (29% vs 42% *P*<0.0001) and average pain severity (28% vs 44% *P*<0.0001) at week 13, but not week 25. Only the comparison on pain interference progression retained statistical significance in favor of enzalutamide at week 25 (23% vs 29% *P*=0.0195).^52^ Median times to progression in BPI-SF worst pain, average pain severity, and pain interference were significantly longer in the enzalutamide arm than placebo arm (Table 4).^52^

#### Radium-223 dichloride

The ALSYMPCA trial compared the effects of radium-223 dichloride with placebo in mCRPC patients with symptomatic bone metastases who had either received docetaxel or were not planning to receive it.^50^ A unique aspect of this trial was that palliative external beam radiotherapy could be administered and patients could also take standard hormonal therapies, such as androgen receptor antagonists or ketoconazole. There was less deterioration in mean FACT-P total score from enrollment to week 16 in the radium-223 dichloride arm than the placebo arm (−2.7 vs −6.8; *P*=0.006). Clinically meaningful improvements in FACT-P total score also favored radium-223 dichloride over placebo (25% vs 16% *P*=0.02; Table 3). In a smaller dose-finding study of 100 patients with painful bone metastases, 56% achieved pain palliation (using the pain index) at 8 weeks after receiving radium-223 dichloride at the approved dose of 50 kBq kg^−1^ monthly.^47^ For patients with pain response in the 50 kBq kg^−1^ dose group, BPI pain severity index decreased from 4.9 at baseline to 3.0 at week 8 (difference, 1.9; *P*=0.002).^47^

#### Sipuleucel-T

The IMPACT trial studied the role of the immunotherapy sip-T in the treatment of mCRPC patients with an expected survival of ⩾6 months.^26^ The original study design specified time to disease-related pain as a coprimary endpoint (along with objective disease progression), but this endpoint was eliminated at enrollment.^46^ Results were released summarizing time to development of disease-related pain (that is, pain post-enrollment) in a *post hoc* pooled analysis of three randomized phase III trials of sip-T in men with asymptomatic or minimally symptomatic mCRPC with an expected survival of ⩾3 months.^46^ Time to disease-related pain was not significantly prolonged in the sip-T arm (Table 4) but time to opiate analgesic use was extended (12.6 months for patients in the sip-T arm vs 9.7 months in the control arm; HR, 0.76; 95% CI, 0.58–0.98; *P*=0.038).^46^ The HR for time to disease-related pain in the IMPACT study was 0.80 (95% CI, 0.56–1.15). ^46^

#### Cabazitaxel

The TROPIC trial examined the role of cabazitaxel in the treatment of mCRPC in the post-docetaxel setting, randomizing patients to either cabazitaxel plus prednisone or mitoxantrone plus prednisone.^33^ The pain response rate (7.7% vs 9.2% *P*=0.63) and median time to pain progression (not reached vs 11.1 months; HR, 0.91; 95% CI, 0.69–1.19; *P*=0.52) using the McGill–Melzack present pain intensity instrument (Table 2) was similar in the mitoxantrone and cabazitaxel treatment groups, respectively. A more recent publication reported no meaningful differences seen in pain palliation between cabazitaxel and mitoxantrone.^48^

#### Docetaxel and estramustine

A phase II trial in Italy randomized 95 mCRPC patients to either docetaxel plus estramustine or docetaxel alone. There were no significant changes from baseline in EORTC QLQ-C30 total scores in either arm during treatment; however, only 59 of 95 patients completed both baseline and first post-treatment questionnaires at week 6, limiting the conclusions that can be drawn from this data set.^49^ At this time point, 15 of 59 patients (25%) receiving either docetaxel alone or with estramustine had an improvement in their pain as measured by EORTC QLQ-C30, and 20% had an improvement on the more detailed BPI.^49^

#### Nontaxane-based chemotherapy

A phase II study looked at the palliative benefit of nontaxane chemotherapy in patients who had progressed on docetaxel.^51^ Patients were randomized to receive either mitoxantrone, vinorelbine or etoposide. The primary endpoint was palliative benefit rate, defined as pain control without disease progression; HRQoL was a secondary endpoint and was measured with EORTC QLQ-C30 plus EORTC QLQ-PR25 (Table 2). In the mitoxantrone arm, palliative benefit rate was 36% vs 20% in the vinorelbine and etoposide arms, although no dedicated pain instrument was used. The authors reported that HRQoL responses were similar for the three groups and that fatigue had improved or stabilized in 24% and 25% of patients, respectively.^51^

## Discussion

In the era of expanded therapeutic options, understanding how new treatments for mCRPC impact health states becomes increasingly important. Among the agents discussed in this review, abiraterone acetate plus prednisone, enzalutamide, and radium-223 dichloride offer clear HRQoL benefits and pain mitigation in addition to extended survival and improved cancer control.^24, 25, 28, 29, 30, 31, 50^ Conversely, the trials of mitoxantrone, docetaxel and cabazitaxel did not find statistically significant differences in HRQoL or pain versus comparison arms.^33, 48, 49, 51^ Sip-T appears to offer no palliative effect but may delay time to first opioid use.^26, 46^

We now have a far better basis for explaining to patients with mCRPC what they can expect as they initiate a new systemic therapy. For patients who have recently progressed to castration resistance and are either asymptomatic or minimally symptomatic, oral hormonal agents abiraterone acetate plus prednisone and enzalutamide offer a means to delay deterioration in HRQoL (FACT-P total score) by ≈4 months^57^ and 6 months,^52^ respectively, relative to their respective controls. The comparator-adjusted delay in mean pain intensity progression (BPI-SF) was 8 months with abiraterone acetate plus prednisone^57^ and the placebo-adjusted delay in deterioration of the FACT-P PCS pain-related score was 6 months with enzalutamide.^52^ Aside from delaying cancer-related symptoms associated with disease progression, these agents are associated with postponement of cytotoxic chemotherapy and its attendant toxicities. For men with symptomatic osseous metastatic disease, radium-223 dichloride offered HRQoL improvement and appeared to slow the subsequent decline in HRQoL when added to standard therapy.

Direct comparisons of the palliative effects and HRQoL impacts of the new agents for mCRPC treatment are not possible, as no head-to-head studies have been performed. One clinical issue that further complicates comparison of HRQoL data is the role of corticosteroids. Among agents currently used in mCRPC, corticosteroids are given routinely or required in conjunction with docetaxel, abiraterone acetate, cabazitaxel, and mitoxantrone, but are not required with enzalutamide or radium-223 dichloride. It is noteworthy that median time to mean pain intensity progression on the BPI-SF among chemotherapy-naive patients who received placebo plus prednisone in COU-AA-302 was 18.4 months, whereas in PREVAIL it was 5.5 months among patients who received placebo only. Other interstudy methodological factors, such as different methods by which a progression was defined and analyzed, likely explain such a large difference in this pain metric. Corticosteroids have known palliative effects but are associated with some risks that may impact HRQoL, including development of thrush, insomnia and fat deposition.^59^ The overall impact of corticosteroids remains poorly defined, especially as many corticosteroid-related toxicities are related to cumulative doses and duration of corticosteroid use increases as life-extending therapies are applied earlier in the treatment paradigm. Thus, careful consideration of the risk-benefit ratio of corticosteroids in mCRPC patients is appropriate; however, we found little exploration of independent effects of corticosteroids on HRQoL and pain palliation in the publications we reviewed.

Contextualizing HRQoL and pain palliation results is made difficult because of the inconsistency in methodology for evaluating these parameters. Most HRQoL instruments commonly used in mCRPC trials were not designed specifically for an mCRPC population. Consequently, these instruments may focus on issues more relevant to prostate cancer patients treated for localized disease and may not capture all major factors that drive HRQoL for men with metastatic disease. For example, Eton *et al.*^60^ used patient interviews to generate a list of 16 issues and outcomes that are most important to mCRPC patients, and included items such as PSA anxiety, urinary obstruction/frequency, and change in self-image, which are not well represented in FACT-P and EORTC QLQ-30 questionnaires. Lack of consistency in applying PRO instruments creates further variability. For example, COU-AA-301 and AFFIRM both used the FACT-P and BPI-SF instruments, but the study methods differ in areas such as frequency of PRO assessments and definition of outcomes. In COU-AA-301, patients were only considered eligible for improvement in HRQoL if their baseline FACT-P total score was ⩽122, whereas in AFFIRM, all patients were considered eligible for improvement in HRQoL regardless of baseline FACT-P score. In addition to establishing standard time points and ‘response' or ‘progression' benchmarks, reporting time-linked outcomes, such as time to pain palliation or duration of pain palliation, and analyzing symptomatic and asymptomatic patient populations separately are needed in order to produce more clinically relevant data.

Another difficulty in understanding the impact of therapies on disease burden and HRQoL in mCRPC comes from translating clinical trial data into real-world practice, as confounding issues relating to study design, patient selection, therapeutic implementation and healthcare delivery contribute to an efficacy-effectiveness gap. Patients enrolled in clinical trials may be healthier than average mCRPC patients, with fewer of the medical comorbidities often found in an older patient population. Patients with mCRPC in clinical trials may also have a lower burden of illness over the course of their disease. Sullivan *et al.*^61^ observed a cohort of 280 mCRPC patients for up to 9 months in the clinical practice setting and found that their deterioration in HRQoL was more rapid than that described in major clinical trials, suggesting that clinical trial data may underestimate HRQoL challenges faced by real-world mCRPC patient populations. This also implies that the quality and depth of HRQoL data collected in a trial depends heavily on the approach used to gather it. More prospective observational data with serial HRQoL assessments is required to elucidate disparities between clinical trial and real-world settings regarding patient well-being. Although HRQoL instruments can be incorporated into practice,^62^ the manner in which HRQoL data from clinical trials are presented must be better standardized and reported in a way that lends itself to incorporation into everyday practice.

Validating the implementation of PRO questionnaires in the clinical practice setting is required to ensure that information is captured accurately and without bias. Yet systematic collection of PRO data in routine clinical practice requires time and effort, placing a burden on patients, families, and clinical staff. In addition, although HRQoL instruments such as FACT-P and EORTC QLQ-C30 are validated for use in research, their utility in general clinical practice is unclear. Ultimately, once a practice completes PROs, clinicians will need tools to view sequential HRQoL information in parallel with the rest of the medical record so that these data can be interpreted in the context of the patient's treatment plan and cancer control. Applications that help clinicians explain to patients the impact of therapy on HRQoL, ideally with graphical depiction, will be helpful in stimulating dialog about the overall value of treatment.

## Conclusion

Since 2010, mCRPC has seen an increasing number of therapeutic options. When considering a specific treatment decision, a clinician must balance the potential HRQoL improvement that could result from disease control with potential HRQoL decrements related to adverse effects associated with treatment. To give context to the relative impact of treatments on HRQoL and pain, it is critical to understand the underlying disease burden in mCRPC patients and to standardize methods for measuring and quantifying HRQoL and symptom assessments. Active treatment with noncytotoxic agents, abiraterone acetate plus prednisone and enzalutamide, and radium-223 dichloride and sip-T, is associated with varying levels of improvement in HRQoL and pain status, but direct comparisons between treatments are not possible. As patients progress to mCRPC and receive life-extending therapies, PROs that are subject to validation processes in the clinical practice setting will be required to monitor their experiences with the disease and its treatment.

## Figures and Tables

**Table 1:** Study-level details of the analyses describing PROs of routinely used agents tested in randomized, controlled trials of patients with metastatic castration-resistant prostate cancer from 2010 to 2015

*Agent*	*Citation*	*Study*	*Interventions*	*No. and type of patients*	*PRO*^a^	*Completion dates*
Abiraterone acetate (AA)	54	COU-AA-301	Daily AA+prednisone (PRED) vs placebo (PBO)+PRED	1195 Post-docetaxel	FACT-P	Baseline and on day 1 of mo 1, 4, 7, and 10 and every 6 mo thereafter until treatment discontinuation
	56				BPI-SF	Baseline, day 15 of mo 1, and day 1 of every subsequent mo
	55				BFI	Baseline, and on the first day of each mo until treatment discontinuation
	29	COU-AA-302	Daily AA+PRED vs PBO+PRED	1088 Pre-docetaxel	FACT-P	Baseline, day 1 of mo 3, 5, 7, and 10, every third mo thereafter, and at end of treatment
	57 28				BPI-SF	Baseline, at day 1 of each mo, and at end of treatment
	31					
Enzalutamide (ENZA)	30	AFFIRM	Daily ENZA vs PBO	1199 Post-chemotherapy	FACT-P	Wk 1, 13, 17, 21, and 25, then every 12 wk while patients were on treatment
					EQ-5D^b^	Baseline, wk 1, 13, 25, and every subsequent 12 wk
	58 35				BPI-SF	Baseline and wk 13 while on treatment
	24	PREVAIL	Daily ENZA vs PBO	1717 Pre-chemotherapy	FACT-P	Baseline, wk 5, wk 13, and then every 12 wk until drug discontinuation
					BPI-SF	Screening, baseline, and wk 13 and 25
	52				EQ-5D	Baseline, wk 13, and every 12 wk until drug discontinuation
Radium-223 dichloride (Ra-223)	50	ALSYMPCA	6 Injections of Ra-223 vs PBO at 4 weekly intervals	921 Patients with ⩾2 bone and no known visceral metastases	FACT-P	Baseline and wk 16
	47	NA	Single-dose, dose-ranging	100 Patients with painful bone metastases	Pain index^c^	Baseline and wk 2, 4, 8, 12, and 16
					BPI	Baseline and wk 2, 4, 8, 12, and 16
Sipuleucel-T (sip-T)	26 46^d^	IMPACT/D9901/D9902A	3 Injections of sip-T vs PBO at 2 weekly intervals	Patients with an expected survival ⩾3 mo^e^	BPI	Baseline and weekly thereafter
Mitoxantrone	51	GETUG-P02	Mitoxantrone vs vinorelbine vs etoposide	92 Post-docetaxel	EORTC QLQ-C30+QLQ-PR25	Before each cycle and every 3 mo during follow-up
Docetaxel/estramustine	49	NA	Docetaxel+estramustine vs docetaxel	59 Pre-chemotherapy	EORTC QLQ-C30 BPI	Baseline and every 6 wk (that is, every 2 treatment cycles)
						
Cabazitaxel	33 48	TROPIC	Cabazitaxel+PRED vs mitoxantrone+PRED	755 Post-docetaxel	McGill–Melzack PPI	Baseline, every 6 wk during the first 6 mo of follow-up, and every 3 mo thereafter, until documented progression or initiation of other anticancer therapy

Abbreviations: BFI, Brief Fatigue Inventory; BPI-SF, Brief Pain Inventory Short Form; EORTC QLQ-C30, European Organization for Research and Treatment of Cancer Quality of Life Questionnaire C30; FACT-P, Functional Assessment of Cancer Therapy-Prostate; mo, months; NA, not applicable; PPI, present pain intensity; PROs, patient-reported outcomes; PR25, EORTC QLQ prostate-specific module; wk, week.

a All PRO data were analyzed in a prespecified manner with the exception of Small *et al.*^46^

b Information collected at select centers in Europe only.

c Combination of the visual analog scale and analgesic consumption categorized according to the World Health Organization analgesic ladder.^64^

d Pooled analysis of three phase III trials, one of which was IMPACT.

e 428 patients analyzed for time to disease-related pain; 737 analyzed for time to first use of opioid analgesics. IMPACT enrolled 512 patients who had an expected survival of ⩾6 months.

**Table 2:** Features and properties of the validated and accepted questionnaires used to evaluate HRQoL and pain in randomized, controlled trials of patients with metastatic castration-resistant prostate cancer

*Questionnaire*	*Description*	*Outcomes scores*	*No. of items*	*Possible score range*	*Established change threshold*
Functional Assessment of Cancer Therapy-Prostate (FACT-P)	Multidimensional 39-item questionnaire made up of 2 parts: the 27-question FACT-G cancer questionnaire and 12-question PCS.^44^ The FACT-G questions are grouped into 4 QoL subscales: physical well-being, social/family well-being, emotional well-being and functional well-being. A decrease in score indicates a worsening patient status; higher scores indicate better QoL.	Total score^a^ General function subscale^b^ PCS Trial outcome index^d^ Physical well-being Social/family well-being Emotional well-being Functional well-being PCS pain related^e^	39 27 12 26 7 7 6 7 4	0–156 0–108 0–48 0–104 0–28 0–28 0–24 0–28 0–16	6–10 (Cella *et al.*^35^) +4 or −8 (Ringash *et al.*^65^)^c^ 2–3 (Yost *et al.*^66^) 5–9 (Yost *et al.*^66^) 2–3 (Yost *et al.*^66^) 2–3 (Yost *et al.*^66^) 2–3 (Yost *et al.*^66^) 2–3 (Yost *et al.*^66^) 1–2 (Yost *et al.*^66^)
European Organization for Research and Treatment of Cancer Quality of Life Questionnaire C30 (EORTC QLQ-C30)	Cancer-specific questionnaire consisting of 30 items: 24 form 9 multi-item scales covering various aspects of QoL, and the remaining 6 are single-item scales describing different cancer-relevant symptoms. The questionnaire makes it possible to obtain 1 global item (global health) and 5 functional domains; 3 symptom scales (fatigue, pain and nausea/vomiting); 5 single-symptom items; and 1 item concerning the financial impact of the disease. During the scoring procedure, scale scores are calculated by averaging items within scales and transforming average scores linearly into 0–100 scales. Higher scores in the global and functioning scales and lower scores in the symptom scales indicate better QoL.^67, 68, 69^	Global health status/QoL Physical functioning Role functioning Emotional functioning Cognitive functioning Social functioning Fatigue Nausea and vomiting Pain Dyspnea Insomnia Appetite loss Constipation Diarrhea Financial difficulties	2 5 2 4 2 2 3 2 2 1 1 1 1 1 1	0–100 0–100 0–100 0–100 0–100 0–100 0–100 0–100 0–100 0–100 0–100 0–100 0–100 0–100 0–100	10 (Osoba *et al.*^70^) 10 (Osoba *et al.*^70^) 10 (Osoba *et al.*^70^) 10 (Osoba *et al.*^70^) 10 (Osoba *et al.*^70^) 10 (Osoba *et al.*^70^) 10 (Osoba *et al.*^70^) 10 (Osoba *et al.*^70^) 10 (Osoba *et al.*^70^) 10 (Osoba *et al.*^70^) 10 (Osoba *et al.*^70^) 10 (Osoba *et al.*^70^) 10 (Osoba *et al.*^70^) 10 (Osoba *et al.*^70^) 10 (Osoba *et al.*^70^)
EORTC QLQ-prostate-specific module (PR25)	A supplemental prostate-cancer–specific module, consisting of 25 items with 6 multi-item subscales assessing urinary and bowel symptoms, sexual activity and functioning, and adverse effects of treatment. Patients are asked to recall the past week. The item and domain scales range 0–100, with higher scores indicating worse symptoms (urinary, bowel) or higher levels of function (sexual).^71^	Total Urinary Bowel Use of incontinence aids Sexual function Sexual interest and functioning Side-effects of hormonal treatment	25 8 4 1 2 4 6	0–100 0–100 0–100 0–100 0–100 0–100 0–100	Undetermined. Tentatively, a 5−10% change may be clinically significant based on the EORTC QLQ-C30 (Marigwa *et al.*^72^)
EQ-5D	An international, standardized, generic questionnaire for describing and valuing HRQoL.^66, 73^ The population preference-based health state utility score (EQ-5D utility index) and patient's overall health state on a visual analog scale (EQ-5D visual analog scale (VAS)) are reliable and valid for assessing HRQoL in cancer patients.^74, 75^ Higher scores represent better health states.	EQ-5D utility index EQ-5D VAS	5 1	−0.594 to 1 0–100	0.04–0.14 (Pickard *et al.*^76^) 7–11 (Pickard *et al.*^76^)
Brief Fatigue Inventory (BFI)	Analogous to the Brief Pain Inventory (see below), the BFI is a standard, reliable instrument used to assess fatigue quickly in patients with cancer. It is significantly correlated with other validated fatigue questionnaires.^77^	Fatigue severity^f^ Fatigue now Worst fatigue Average fatigue Fatigue interference^g^ General activities Mood Walking Normal work Relationships with others Enjoyment of life	3 1 1 1 6 1 1 1 1 1 1	0–10 0–10 0–10 0–10 0–10 0–10 0–10 0–10 0–10 0–10 0–10	⩾2 (Sternberg *et al.*^53^) ⩾1.25 (Sternberg *et al.*^53^)
Brief Pain Inventory (BPI) and BPI Short Form (BPI-SF)	Self-assessment tool measuring pain intensity and the amount pain interferes with activities of daily living rated using an 11-point numerical scale of 0–10, with 10 being the worst level of pain or interference (‘pain as bad as you can imagine') and 0 being no pain interference (‘no pain').^h^ Each interference item is scored 0–10, with 0 representing ‘does not interfere' and 10 indicating ‘completely interferes.' The most important difference between the longer and shorter versions of the BPI is that the latter uses a 24-h recall period.^45^	Pain severity^i^ Worst pain Least pain Average pain Pain now Pain interference^j^ General activities Mood Walking Normal work Relationships with others Sleep Enjoyment of life	4 1 1 1 1 7 1 1 1 1 1 1 1	0–10 0–10 0–10 0–10 0–10 0–10 0–10 0–10 0–10 0–10 0–10 0–10 0–10	Increase ⩾30% or ⩾2 points^78^ Increase ⩾50% of baseline s.d.
McGill–Melzack present pain intensity (PPI)	The single question about PPI is often used alone as a single scale of 0–5. Patients choose a number between 0 (none) and 5 (excruciating).^79, 80^	PPI^k^	1	0–5	⩾2 (Serlin *et al.*^81^)
Pain Index	Derived from a combination of the VAS and analgesic consumption categorized according to the World Health Organization analgesic ladder.^64^			1–4 5 6	Pain responders No response Pain progression

Abbreviations: FACT-G, Functional Assessment of Cancer Therapy-General; HRQoL, health-related quality of life; PCS, prostate cancer subscale.

The established change thresholds represent clinically meaningful changes.

a Composite of the scores on physical well-being+social/family well-being+emotional well-being+functional well-being+the score on the PCS. Impaired QoL has been defined arbitrarily in the published literature as a FACT-P score of ⩽122^55^ and ⩽128,^82^ of the 156 maximum score.

b Composite of the scores on physical well-being+social/family well-being+emotional well-being+functional well-being.

c A gain of ≈4 points may be a clinically meaningful improvement and a loss of ≈8 points may indicate clinically meaningful deterioration.

d Composite of the scores on physical well-being+functional well-being+PCS.

e Calculated by using the four questions on pain in the FACT-P, but the scores are reversed such that higher score indicates better health and less pain. A decrease in score signifies pain progression.

f Composite of the scores on fatigue now, worst fatigue and average fatigue.

g Composite of the scores on general activity, mood, walking ability, normal work, relationships with others, sleep and enjoyment of life.

h Clinically significant pain on the BPI-SF is defined as a score of ⩾4 on item 3 (pain at its worst in the last 24 h) and a score of ⩾4 on the pain interference scale.^81^

i Composite of the scores on worst pain, least pain, average pain and pain now.

j Composite of the scores on general activity, mood, walking ability, normal work, relationships with others, sleep and enjoyment of life.

k A PPI ⩾2 is considered pain that is at a discomforting or worse level.^83^

**Table 3:** Proportion of patients with metastatic castration-resistant prostate cancer reporting an improvement on the FACT-P total score and FACT-P PCS score after receipt of new, routinely used agents

*Study*	*Type of patients*	*Treatment*	*No. of patients randomized*	*Response rate*, n*/*N*a (%)*	
				*FACT-P total score*	*FACT-P PCS score*
COU-AA-301 (Harland *et al.*^55^)	Post-docetaxel	AA+PRED	797	271/563 (48)^b^	325/554 (59)^b^
		PBO+PRED	398	87/273 (32)	101/255 (40)
COU-AA-302 (Rathkopf *et al.*^28^)	Pre-docetaxel	AA+PRED	546	NR	NR
		PBO+PRED	542	NR	NR
AFFIRM^30, 58^	Post-chemotherapy	ENZA	800	275/652 (42)^b^	366/665 (55)^b^
		PBO	399	36/248 (15)	65/255 (25)
PREVAIL^52^	Pre-chemotherapy	ENZA	872	327/826 (40)^b^	466/843 (55)^b^
		PBO	845	181/790 (23)	275/807 (34)
ALSYMPCA^50^	Pre- and post-docetaxel (patients with ⩾2 bone and no known visceral metastases)	Ra-223 PBO	614 307	(25)^c^ (16)	NR NR

Abbreviations: AA, abiraterone acetate; ENZA, enzalutamide; FACT-P, Functional Assessment of Cancer Therapy-Prostate; PBO, placebo; PCS, prostate cancer subscale; PRED, prednisone; Ra-223, radium-223 dichloride; NR, not reported.

a Denominator represents the number of patients eligible for analysis.

b *P*<0.0001 versus comparator.

c *P*<0.02 versus comparator.

**Table 4:** Median time to pain progression (months) associated with new, routinely used agents for metastatic castration-resistant prostate cancer

*Study*	*No. and type of patients*	*Treatment*	*Instrument*	*Pain severity*		*Worst pain*		*Interference*	
				*Median time to progression*	*Hazard ratio (95% CI)*	*Median time to progression*	*Hazard ratio (95% CI)*	*Median time to progression*	*Hazard ratio (95% CI)*
COU-AA-301 (Logothetis *et al.*^56^)	1195 Post-docetaxel	AA+PRED	BPI-SF	NR	NR	7.4	0.72^a^ (0.56–0.92)	9.3	0.65^b^ (0.50–0.83)
		PBO+PRED		NR		4.8		4.6	
COU-AA-302 (Rathkopf *et al.*^28^)	1088 Pre-docetaxel	AA+PRED	BPI-SF	26.7	0.83 (0.68–1.01)	25.8	0.85 (0.69–1.04)	10.3	0.80^d^ (0.68–0.93)
		PBO+PRED		18.4^c^		20.3		7.4	
AFFIRM^58^	1199 Post-chemotherapy	ENZA	FACT-P item 4^e^	NYR	0.56^f^ (0.41–0.78)	NA	NA	NA	NA
		PBO		13.8		NA		NA	
PREVAIL^52^	1717 Pre-chemotherapy	ENZA PBO	FACT-P PCS pain related	8.3 2.8	0.58^g^ (0.51–0.67)	NA NA	NA	NA NA	NA
		ENZA	BPI-SF	5.6	0.60^e^ (0.51–0.71)	5.6	0.62^g^ (0.53–0.74)	5.8	0.57^g^ (0.48–0.69)
		PBO		5.5^c^		5.5		5.6	
D9901, D9902A, D9902B^46^	428 Patients with an expected survival>6 mo	Sipuleucel-T Control	BPI-SF	5.6 5.3	0.82 (0.62–1.09)	NR NR	NR	NR NR	NR

Abbreviations: AA, abiraterone acetate; BPI-SF, Brief Pain Inventory Short Form; CI, confidence interval; ENZA, enzalutamide; FACT-P, Functional Assessment of Cancer Therapy-Prostate; mo, month; NA, not applicable; NR, not reported; PBO, placebo; PCS, prostate cancer subscale; PRED, prednisone.

a *P*=0.0088 versus PBO.

b *P*=0.0006 versus PBO.

c In COU-AA-302, clinically meaningful progression in mean pain intensity was defined as a >30% increase from baseline in BPI-SF score without decreased analgesic usage score at two consecutive visits,^28^ whereas in the PREVAIL study clinically meaningful progression in mean pain intensity was defined as a >30% increase from baseline in BPI-SF score at any visit.^52^

d *P*=0.005 versus PBO.

e FACT-P item 4 is listed in the physical well-being domain as ‘I have pain.'

f *P*=0.0004 versus PBO.

g *P*<0.0001 versus PBO.
